# Stenosing Tenosynovitis of the Flexor Hallucis Longus Tendon Associated with the Plantar Capsular Accessory Ossicle at the Interphalangeal Joint of the Great Toe

**DOI:** 10.1155/2017/2146762

**Published:** 2017-01-31

**Authors:** Song Ho Chang, Takumi Matsumoto, Masashi Naito, Sakae Tanaka

**Affiliations:** Department of Orthopaedic Surgery, Faculty of Medicine, The University of Tokyo, 7-3-1 Hongo, Bunkyo-ku, Tokyo 113-8655, Japan

## Abstract

This report presents a case of stenosing tenosynovitis of the flexor hallucis longus tendon associated with the plantar capsular accessory ossicle at the interphalangeal joint of the great toe, which was confirmed by intraoperative observation and was successfully treated with surgical resection of the ossicle. As the plantar capsular accessory ossicle was not visible radiographically due to the lack of ossification, ultrasonography was helpful for diagnosing this disorder.

## 1. Introduction

Portions of the general population have a plantar capsular accessory ossicle at the interphalangeal joint (IPJ) of the great toe [1–5]. Although usually asymptomatic, this accessory ossicle sometimes becomes troublesome, causing painful plantar callosities [6, 7], inflammation of the ossicle [8], inflammation of the flexor hallucis longus (FHL) tendon [9], or irreducible interphalangeal joint dislocation due to its interposition [10, 11]. In cases that do not respond to conservative treatment, surgery may be the best treatment option. To the best of our knowledge, this is the first documented case of stenosing tenosynovitis of the FHL among literatures written in English, associated with an IPJ plantar capsular accessory ossicle, leading to pain at the IPJ and significantly reduced IPJ flexion, which was successfully resolved through surgical removal of the ossicle.

## 2. Case Report

**Figure 1:**
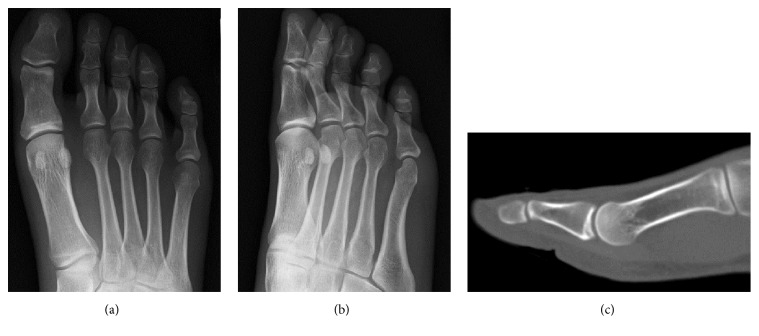
Plain radiographs and computed tomography showed no significant abnormality. (a) Anteroposterior radiograph of the foot. (b) Oblique radiograph of the foot. (c) Sagittal plane of the great toe in computed tomography.

**Figure 2:**
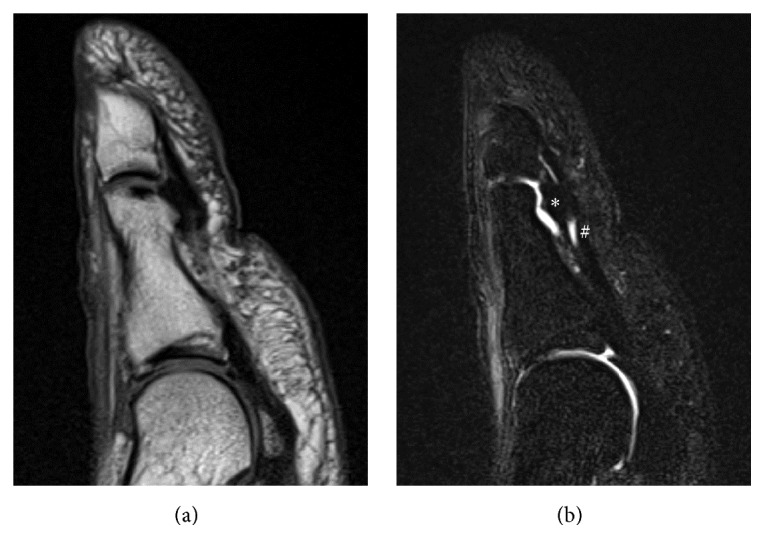
Sagittal T1 and T2 weighted magnetic resonance (MR) images showed a nodule in the plantar capsule at the interphalangeal joint of the great toe and fluid retention around it and the flexor hallucis longus tendon. (a) T1 weighted MR image. (b) T2 weighted MR image. ^*∗*^Plantar capsule nodule. ^#^Flexor hallucis longus.

**Figure 3:**
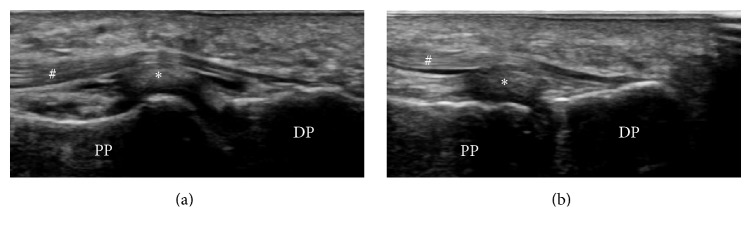
Ultrasonography image of the interphalangeal joint (IPJ) showed fluid retention around the plantar capsule nodule and flexor hallucis longus tendon on the affected side, but not on the healthy side, of the great toe. (a) Affected side. (b) Healthy side. DP: distal phalanx and PP: proximal phalanx. ^*∗*^Plantar capsule nodule. ^#^Flexor hallucis longus.

A previously healthy, 28-year-old male complained of persistent pain at the IPJ and inability to flex the IPJ of the great toe for the past month. One week prior to noticing the loss of IPJ flexion, he had discomfort at the IPJ after exercise, although he had had no apparent episode of trauma. His doctor ordered an MRI, but no definite diagnosis was established at that time. His doctor recommended conservative treatment—including protected weight-bearing and use of anti-inflammatory medication—but this failed to relieve the patient's symptoms. He presented to our hospital two months after the onset of symptoms. On examination, the patient had difficult IPJ active flexion, acute tenderness at the plantar aspect of the IPJ, and pain with passive flexion of the IPJ. Plain radiography and computed tomography (CT) showed no significant abnormality (Figure 1). Magnetic resonance imaging (MRI) showed a single elliptical nodule in the plantar capsule at the IPJ of the great toe, distinct from the FHL tendon, and showed fluid accumulation in the IPJ and the tendon sheath of the FHL indicative of tenosynovitis (Figure 2). Dynamic observation via ultrasonography showed continuity of the FHL tendon from the toe to the ankle, ruling out FHL tendon rupture as a cause of inability to flex the IPJ. Ultrasonography of the plantar aspect of the IPJ revealed fluid retention around the nodule and FHL tendon on the affected side, but not the healthy side (Figure 3). From these observations we suspected that the stenosing tenosynovitis of the FHL was associated with the IPJ plantar capsular accessory ossicle. Local infiltration with 0.5 mL of 1% lidocaine mixed with 10 mg of triamcinolone acetonide had limited effect, only partially relieving the pain for a week. We proposed surgical treatment and the patient agreed.

**Figure 4:**
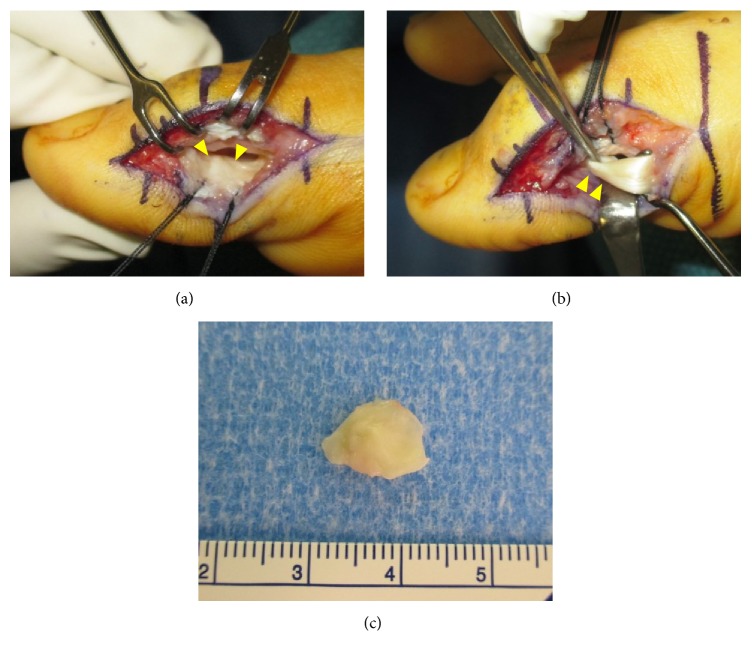
Surgical resection of the plantar capsular accessory ossicle at the interphalangeal joint (IPJ) via medial approach. (a) The IPJ plantar capsular ossicle (arrow heads). (b) The constricted flexor hallucis longus tendon at the level of the IPJ plantar capsular ossicle (arrow heads). (c) Removed IPJ plantar capsular accessory ossicle.

The procedure was performed via a longitudinal 2 cm skin incision over the medial side of the IPJ of the great toe. The digital nerve was identified and retracted below the surgical field. The capsule was opened and a firm round nodule approximately 1 cm by 1 cm was seen to be embedded in the plantar capsule. The nodule had the cartilaginous appearance at its intra-articular aspect (Figure 4). The FHL ran through the tendon sheath as part of the plantar capsule of the IPJ. The FHL tendon was constricted beneath the tendon sheath so tightly that we had difficulty passing the tip of a small bone elevator between the tendon and tendon sheath. We opened a 3 mm length of the tendon sheath from its proximal end and visualized the FHL tendon at the level of the nodule (Figure 4). The nodule was detached from the capsule using surgical scissors. The procedure did not damage the plantar capsule in any way. The FHL tendon could slide easily once the nodule was removed. No postoperative external fixation was used. Postoperative recovery was uneventful. Follow-up six months later found the patient had returned to normal activity without any pain or functional impairment.

## 3. Discussion

The reported incidence of the ossicle at the plantar aspect of the hallucial IPJ varies widely, from 2% to 96% [1–5]. A possible explanation for this extreme difference in reported rates is the difference in observational methods between plain radiography and studies of cadavers. Racial and geographical variation may also cause discrepancies in observed rates. The accessory ossicle was found in 96% of Japanese cadavers [3], but in only 73% of British Caucasian cadavers [12]. Radiographic observation found the ossicle in 91% of Japanese subjects [3], 86% of Thai subjects [13], 13% of North American subjects [14], and only 2% of Turkish subjects [5].

This condition can result in several different clinical presentations including painful plantar callosities [6, 7], inflammation of the ossicle and/or FHL tendon [8, 15], and irreducible IPJ dislocation due to the interposition of the ossicle [10, 11]. However, to our knowledge, stenosing tenosynovitis of the FHL by the plantar capsular accessory ossicle presenting with limited IPJ flexion has not been previously reported.

A sesamoid is a bone, embedded within a tendon, which serves to modify pressure and alter the direction of muscle forces [2]. In earlier literature the term sesamoid was used to describe the ossicle at the plantar aspect of the IPJ, because it was assumed that the ossicle lies within the fibers of the FHL [16]. Later, better defined dissection studies proved that, in fact, the ossicle lies within the plantar capsule and therefore the term accessory ossicle or intra-articular ossicle was proposed [12, 13]. The theory that this ossicle is derived from a rudiment of the lost middle phalanx of the great toe is generally accepted [17].

Diagnosing IPJ plantar capsular ossicle disorder is often difficult. The ossicles are easily overlooked on radiography, especially in cases with incomplete ossification. Because they are small, oval, rough, and convex in shape, their contours on radiographs often are obscured by the opacity of the phalanges [12, 13, 15]. Ultrasonography and MRI are both useful tools for detecting small cartilaginous nodules that cannot be detected with radiography [8, 15]. However, ultrasound is superior to MRI in that it can provide dynamic anatomical information of both the ossicle and FHL tendon.

Conservative treatment of a symptomatic IPJ plantar capsular accessory ossicle of the great toe includes rest, use of a pad for decompression, shaving of hyperkeratotic lesions, and local corticosteroid injection [2, 15]. In cases where conservative treatment is ineffective, surgical removal should be considered [9]. In our case intractable pain and persistent IPJ flexion disorder was unresponsive to conservative treatment but the removal of the IPJ plantar capsular ossicle relieved both symptoms completely. This result demonstrated that the symptoms were caused by irritation of the FHL tendon related to pathology of the IPJ plantar capsular ossicle.

A number of procedures for surgical removal of the accessory ossicle are in use [16–19]; they are generally divided into medial, plantar, and dorsal approaches. Each has distinct advantages and disadvantages over the others. The plantar approach provides the most direct approach to the ossicle and offers the possibility of concomitant resection of plantar hyperkeratotic lesion; however, this approach is frequently complicated by residual hypertrophic scar tissue formation. The dorsal approach is often used in cases of ossicle interposition associated with IPJ dislocation [11]; however, extensive invasions—including tenotomy, capsulotomy, and collateral ligament release—are required to achieve complete exposure of the ossicle in nondislocated cases [16]. The medial approach creates an incision extending from just distal to the first metatarsophalangeal joint to the base of the distal phalanx of the great toe. With a plantarflex position of the IPJ, the FHL is relaxed and allows direct visualization of the plantar ossicle. This approach provides the least surgical exposure, avoids neurovascular bundles, and does not cross lines of flexion and extension of the great toe [6, 17]. These are significant advantages since they prevent painful hypertrophic scar tissue formation in a weight-bearing area. For these reasons, we chose the medial approach, and we had no postoperative complications.

## 4. Conclusion

We conclude that stenosing tenosynovitis of the FHL tendon associated with the IPJ plantar capsular accessory ossicle should be taken into consideration in the differential diagnosis of IPJ flexion disorder. Further, cases unresponsive to conservative treatments may benefit from surgical removal of the ossicle.
